# Commitment of Oral-Derived Stem Cells in Dental and Maxillofacial Applications

**DOI:** 10.3390/dj6040072

**Published:** 2018-12-13

**Authors:** Gianrico Spagnuolo, Bruna Codispoti, Massimo Marrelli, Carlo Rengo, Sandro Rengo, Marco Tatullo

**Affiliations:** 1 Department of Neurosciences, Reproductive and Odontostomatological Sciences, University of Naples “Federico II”, 80131 Naples, Italy; carlorengo@alice.it (C.R.); sanrengo@unina.it (S.R.); 2 Unit of Experimental Medicine, Marrelli Health, 88900 Crotone, Italy; bruna.codispoti@tecnologicasrl.com (B.C.); prof.marrelli@libero.it (M.M.); 3 Tecnologica Research Institute, Stem Cells Unit, 88900 Crotone, Italy; marco.tatullo@tecnologicasrl.com

**Keywords:** oral-derived stem cells, regenerative medicine, bone tissue, tissue engineering

## Abstract

Tissue engineering is based on the interaction between stem cells, biomaterials and factors delivered in biological niches. Oral tissues have been found to be rich in stem cells from different sources: Stem cells from oral cavity are easily harvestable and have shown a great plasticity towards the main lineages, specifically towards bone tissues. Dental pulp stem cells (DPSCs) are the most investigated mesenchymal stem cells (MSCs) from dental tissues, however, the oral cavity hosts several other stem cell lineages that have also been reported to be a good alternative in bone tissue engineering. In particular, the newly discovered population of mesenchymal stem cells derived from human periapical inflamed cysts (hPCy-MSCs) have showed very promising properties, including high plasticity toward bone, vascular and neural phenotypes. In this topical review, the authors described the main oral-derived stem cell populations, their most interesting characteristics and their ability towards osteogenic lineage. This review has also investigated the main clinical procedures, reported in the recent literature, involving oral derived-MSCs and biomaterials to get better bone regeneration in dental procedures. The numerous populations of mesenchymal stem cells isolated from oral tissues (DPSCs, SHEDs, PDLSCs, DFSCs, SCAPs, hPCy-MSCs) retain proliferation ability and multipotency; these features are exploited for clinical purposes, including regeneration of injured tissues and local immunomodulation; we reported on the last studies on the proper use of such MSCs within a biological niche and the proper way to storage them for future clinical use.

## 1. Introduction

Stem cells have attracted a growing interest in the scientific community mainly for their ability to regenerate damaged tissues and also for their potential in modulating inflammatory and immune responses.

These skills have caused stem cells to be considered as a strategic tool for a large number of clinical applications; this is demonstrated by the increasing number of clinical trials focused on stem cell therapy that nowadays amounts to about 7000 studies found for “stem cells” as searching criteria on ClinicalTrials.gov website.

The elevated proliferation potential and the wide differentiation ability of mesenchymal stem cells (MSCs) strongly encourages scientists to search for new unexplored sources of these precious cells, however, their extreme plasticity and their ability to modify different biological niches has caused some scientists to still have doubts about their own existence, carrying out alternative hypotheses on MSCs real nature [1]. 

Dental tissues are rich in cell populations expressing mesenchymal stem cell like-features, with the advantage of having easier access to the oral cavity than other anatomical sites, and the fact that in vitro isolation of cells is very efficient; thus, oral-derived MSCs are highly attractive for research and clinical purposes [2]. Several studies have investigated into oral-derived stem cells used in the regulation of the immune response associated to tissues and organs transplants. Stem cells harvested from dental pulp have been shown to induce immunologic tolerance and to prevent alloreactivity associated with solid-organ or hematopoietic allogeneic transplantation by suppressing T-cell proliferation [3]. 

The immunomodulatory activity of oral-derived MSCs was specifically tested in experimental treatments of autoimmune diseases; for example, stem cells from exfoliated deciduous teeth were capable of promoting the recovery of the ratio between T-reg and T-helper cells with the result to efficiently reduce systemic lupus erythematosus-associated disorders [4]. 

In vitro investigations demonstrated that dental pulp stem cells can stimulate angiogenesis/vasculogenesis, thus finding applications for the cell-based treatments of ischemic diseases [5]. 

Oral stem cells have embryological development from neural crests, and these cells likely maintain a kind of “neural genetic memory”; this hypothesis is supported by the presence of basal gene-expression of neurogenic markers of these cells. The neurogenic potential of oral-derived stem cells has been more consistently demonstrated in the last discovered human periapical-cyst Mesenchymal Stem Cells (hPCy-MSCs) isolated from the inner layer of an inflamed periapical cyst [6]. 

Literature have consistently confirmed that dental stem cells can be easily stimulated to differentiate into osteoblasts and into odontoblasts; this ability make them have a central role in regenerative dentistry [7]. 

The aim of this narrative review is to assess the state-of-the-art and main future insights of oral-derived MSCs; we strongly suggest to use specific oral-derived MSCs as a strategic tool for dental and maxillofacial applications in future MSCs-based therapies.

### Literature Searching Strategy

A literature search was performed through Medline, via PubMed and Google Scholar, to identify articles published from 2000 until 2018. The following keywords have been used for the literature search: “MSCs AND Dental”, “Regeneration AND Dental”, “Bone AND Dental”. The authors have carefully revised both the title and the abstract for each of the selected papers; only papers related to our aim were included in the current narrative review. 

After having excluded the duplicate articles and other types of publication, a total of 571 articles were selected for the current review. Three-hundred-and-sixty-eight articles were considered unsuitable because titles were not fitting the objectives of our review. After an initial screening, a total of 91 papers were excluded. Forty papers were also excluded, since their conclusions were considered not relevant to our aims. Five further papers were excluded, mainly because they revealed methodological bias and a poor reliability of the research. Thirty-five articles met the final inclusion criteria of this narrative review: They were included in this review and we based our main assessment on the base of their scientific messages. 

## 2. Discussion

Stem cell therapy plays a central role in regenerative medicine, with many examples of progression from the preclinical to the early-clinical levels, for a wide variety of diseases.

The first adult stem cells discovered in bone marrow were hematopoietic progenitors able to reconstitute the entire blood system of an immunocompromised host [8] just a few years later. *Friedenstein* first described a population of “clonal, plastic-adherent cells” residing in the blood tissue [9]. These cells were able to self-renew like hematopoietic stem cells, and they possessed the ability to differentiate into the three main stromal layers: fat, bone and cartilage. This population was named Mesenchymal Stem Cells (MSCs) and was phenotypically defined by the expression of specific surface markers: CD105, CD90, CD73 and a lack of markers typically expressed by hematopoietic cells: HLA-DR, CD45 and CD34 [10].

Bone marrow was initially considered the main source of MSCs. Subsequently, it will be discovered that these cells reside not only in bone marrow but also in many other anatomical sites, such as: blood, cord blood, fat, lung, heart, brain, skin, muscle, bowel, liver, gonads, and teeth [11,12].

Furthermore, a series of studies demonstrated that these cells could choose among several further differentiating lineages including skeletal muscle, tendon and neural commitment; these data demonstrated that MSCs hold high plasticity [13,14]. The capability of MSCs to differentiate into several cell types, as well as their important immunomodulatory effects, make them an attractive therapeutic tool for a regenerative medicine purpose, including cell transplantation and tissue engineering. 

### 2.1. Oral-Derived Mesenchymal Stem Cells

Collection of MSCs from human bone marrow (hBM) does not imply a quite simple procedure, indeed, the donor must undergo an invasive intervention to allow aspiration of BM from the iliac crests. Moreover, isolated cells are not abundant because of the low frequency of MSCs in BM estimated to be nearly of one MSC per 34,000 nucleated cells [15]. These issues addressed the interest of researchers toward alternative sources of precious MSCs, in order to obtain a major number of cells and mainly to reduce patient morbidity.

The discovery of ubiquitary cells carrying the typical features of MSCs in the oral cavity, strongly shifted the scientific attention on dental tissues. Oral stem cells can be easily isolated from teeth extracted for orthodontic or irreversible periodontitis reasons. The easy accessibility to the collection site and the abundance of highly immature cells are certainly attracting hallmarks for stem cell therapy studies. Over time, different investigations have shown the existence of a growing number of stem cell populations, with the typical features of MSCs, in oral tissues. 

In 2000, Gronthos first showed the existence of odontogenic progenitors in adult human dental pulp, which were capable of self-renewing with a high proliferative rate and were able to form colonies in in vitro experiments. Moreover, this population was able to reproduce dentin/pulp-like structures after transplantation into immunocompromised mice. These odontogenic precursors shared the same immunophenotype of bone marrow stromal cells (BMSCs) and were finally named dental pulp stem cells (DPSCs) [16]. Thus, DPSCs reflect the overall peculiarities of adult stem cells, including a wide plasticity, demonstrated by several subsequent investigations describing the possibility to differentiate these cells not only into osteocytes, chondrocytes and adipocytes but also into hepatocytes [17] myocytes [18], neurons [19] and hair follicle cells [20].

Three years after the discovery of DPSCs, Miura and colleagues isolated multipotent, clonogenic and highly proliferating progenitors from the dental pulp of human exfoliated deciduous teeth (SHEDs); these cells have shown the ability to in vitro differentiate into various cell types such as odontoblasts, adipocytes and neural cells and to promote dentin formation and bone regeneration in vivo; in addition, their neural marker’s expression allows them to survive in mouse brains after transplantation [21].

A population expressing mesenchymal stem cell-like markers was found to be located in the connective tissue that bonds cementum to alveolar bone, periodontal ligament (PDL). Periodontal ligament stem cells (PDLSCs) can differentiate into adipocytes, collagen-forming cells, and cementoblast-like cells in culture and regenerate cementum and PDL in vivo [22].

The connective sac that surrounds the developing tooth, defined as dental follicle, contains MSCs called dental follicle progenitors (DFPCs); these cells isolated for the first time by Morsczeck in 2005 were able to begin neural differentiation and to regenerate periodontal and bone tissues [23].

Root apical papilla of human teeth holds stem cells (SCAP) able to differentiate into osteogenic and odontogenic progenitors, and also into adipose and neural cells [24,25].

Also, gingival tissues have been shown to be colonized by a multipotent clonogenic stem and progenitor cells. MSCs derived from gingiva, removed for hyperplastic or aesthetic reasons, displayed regenerative potential, for cell therapy, even superior to BMSCs [26].

Progenitors able to differentiate into chondrocytes and osteoblasts are located in the jaw periosteum [27]. Human parotid glands contain cells expressing both epithelial and mesenchymal-specific markers and are able to form colonies in culture [28].

### 2.2. The Human Periapical Cyst-Mesenchymal Stem Cells (hPCy-MSCs)

The oral cavity is certainly a complex environment where many biological niches hosting MSCs have been found in both hard tissues and soft tissues. Scientists commonly recognize Gronthos as the first isolated MSCs population from dental tissues; in fact, he first reported about MSCs from dental pulp (DPSCs). DPSCs have been considered in the last few decades as a good substitute of bone marrow stem cells (BMSCs) for dental applications, because of their ability to induce dentinogenesis in immunocompromised mice. 

In 2013, a new population of mesenchymal stem cells was isolated from human periapical cysts. Tatullo and his team demonstrated for the first time the existence in periapical inflamed tissues of residing cells showing the representative features of MSCs that have been termed: human Periapical Cyst-Mesenchymal Stem Cells (hPCy-MSCs) [29]. This new stem cell cytotype inside the inner layer of the periapical inflammatory cyst wall has the characteristic to be easily isolated from a discarded tissue, usually considered as biological “waste”. The ductility of hPCy-MSCs makes them perfect candidates for bone regeneration. DPSCs and hPCy-MSCs are two MSCs lineages isolated from the oral cavity, with a strong commitment towards osteogenesis and dentinogenesis. In the recent investigations on these new MSCs, it has been demonstrated that hPCy-MSCs have phenotypic and molecular characteristics, as well as differentiating properties, very similar to other well-known MSCs; more specifically, they are: CD13+, CD29+, CD44+, CD73+, CD90+, CD105+, CD45−, stro1+ and CD146+. Despite the expression of several mesenchymal stem cell surface markers seems to stay more stable in cell culture, whereas CD146 expression rapidly declined during passaging. Furthermore, two sub-populations of hPCy-MSCs with different CD146 expression levels have been sorted as CD146Low and CD146High respectively. The differential phenotypical expression of CD146 has been shown to influence the cellular behavior; In fact, CD146Low cells showed an increased proliferation rate, exhibited higher colony-forming unit-fibroblast ability, displayed a greater osteogenic potential, and expressed higher levels of the stemness gene Klf4 when compared to the CD146High subset. Thus, the reducing expression of CD146 seems to be related to a decrease in the differentiation potential and proliferative ability of hPCy-MSCs [30].

hPCy-MSCs in their basal state express high levels of transcripts for neuronal markers and for neural-related transcription factors with respect to dental pulp stem cells. Moreover, this predisposition toward the neural phenotype significantly improves under neurogenic stimulating conditions, as demonstrated by the up-regulation of a broad set of proteins and genes that define neuronal cells [6].

Preliminary data evidence shows the ability of hPCy-MSCs to differentiate into functional dopaminergic neurons, with important potential applications in the treatment of Parkinson’s disorders (Tatullo unpublished). 

In a comparative study, the potential to differentiate into osteoblasts and into odontoblast was investigated between DPSCs and hPCy-MSCs; these populations were cultured in standard conditions and also in osteogenic inducing medium. The obtained results elucidate the more pronounced tendency of DPSCs to undertake the odontoblastic differentiation lineage instead of hPCy-MSCs with a major propensity toward osteogenesis. These data sustain the important value of these cells for dental and maxillofacial regenerative applications [7]. 

Finally, the presence of untreated pulp-periodontal infection does not preclude the possibility of finding resident DPSCs: This could indicate that hPCy-MSCs acts as a co-factor in the process of local bone regeneration. Instead, DPSCs have commonly showed a greater ability to shift towards the odontogenic phenotype. hPCy-MSCs exert extensive proliferative ability and have shown the potential to differentiate into various cell types such as adipocytes, osteoblasts and neurons. The great advantage in the use of this newly discovered oral stem cell population consists in its easy collection from the surgically-removed periapical cysts that imply the reuse of biological waste that became a very smart source of stem cells without any impact on the surrounding healthy tissues [31]. Thus, hPCy-MSCs could be considered an innovative source for stem cell therapy applications.

### 2.3. Tissue Engineering and Regenerative Medicine (TERM)

Mesenchymal stem cells retain a central role in regenerative medicine because of their intrinsic self-renewal ability and their multipotency that allows them to differentiate into both mesenchymal and non-mesenchymal lineages [32]. 

MSCs have demonstrated to be obtainable from stem cell niches that are widely represented in the human body, as well as from somatic cells induced to show MSC-like behavior through genetic manipulations: the iPS (induced pluripotent stem cells) have been widely studied in several therapeutic applications, thanks to their easiness to be obtained from any tissue. 

Recently, reprogrammed iPS were transplanted into the brain of Parkinson’s disease patients, showing how the reprogramming of cells towards an embryonic-like state may be an effective strategy to strengthen the clinical use of MSCs–based therapies [33].

MSCs residing in the oral cavity tissues receive clinical interest in oral and maxillofacial surgery as a cell source that is easy to obtain, easy to manage in vitro and has an excellent behavior after they undergo several differentiating media, even if they have clearly shown a better commitment towards neurogenic, bone and dental tissues such as cementum, dentin and periodontal ligament [2]. 

Furthermore, recent papers suggest the isolation of cellular-secreted nanoparticles that include numerous patterns of functional molecules physiologically used for cell-to-cell cross talking. The possibility to isolate and to subsequently cryo-store these nanovesicles represents a further advantage for the use of the product of stem cells avoiding some existing concerns about the clinical use of stem cells [34].

The presence of a scaffold constituted by biocompatible materials, alone or associated with stem cells, represents a fundamental element in tissue engineering applications. Recent studies elucidate the important role of scaffolds, considered not only as a merely inert support for seeded cells, but a determinant substrate able to affect cell behavior. This concept is applicable to DPSCs, in fact, in their physiological niche, besides biochemical factors, these cells are influenced by biophysical and biomechanical stimuli, such as mechanical stretch, tensile strain and micro- and nano-scale surface topography interaction. DPSCs are sensitive to mechanical forces; these signals are converted into functional cellular responses including lineage specification [35].

## 3. Conclusions

This narrative review has investigated about the state-of-the-art of oral-derived MSCs and on their commitment towards the most useful lineages in dental and maxillofacial applications, with special attention to bone regeneration. MSCs in regenerative medicine are still an open challenge: their use must become applicable in the daily reconstructive surgery. In this review, with particular focus on osteogenic commitment, we found that all MSCs from oral tissues were highly committed to differentiate toward osteoblasts and precursors of bone tissue. Interestingly, hPCy-MSCs showed a better suitability towards osteogenic commitment, whereas DPSCs appeared to be more direct towards dentinogenesis, even if both these cell lineages were able to achieve good results in experimental sessions related to differentiation in the above cited lineages. The authors feel that hPCy-MSCs could be a good alternative to MSCs from other sources, given the reduced ethical and biological issues affecting this specific novel MSC lineage. Different trials are ongoing in a clinical setting, however, future investigations are needed to better understand the biology of oral-derived MSCs, including further undiscovered differentiation fates, and to explore more in depth the potentialities of the secretome of these cells, aiming to reach the final goal of shifting the results from the research laboratory to patients. 

## Abbreviations

DPSCs	Pulp Stem Cells
SHEDs	Human Deciduous Teeth Stem Cells
PDLSCs	Periodontal Ligament Stem Cells
DFSCs	Dental Follicle Stem Cells
SCAPs	Stem Cells from Apical Papilla
hPCy-MSCs	human Periapical Cysts-Mesenchymal Stem Cells
